# Supplementary material for: Complexome profiling of the Chlamydomonas psb28 mutant reveals TEF5 as an early PSII assembly factor
Source: Plant Cell. 2025 Mar 17;37(6):koaf055. doi: 10.1093/plcell/koaf055 (PMC12164590; doi:10.1093/plcell/koaf055)
Supplement: koaf055_Supplementary_Data [file koaf055_supplementary_data.zip › psb28_interactive.zip!/plots/intensity/qualityPlots/peptides/PRPL4_Cre11.g479500.t1.2.html]

### Cre11.g479500.t1.2

Trivial name: PRPL4  
SeqCoverage [median]: 19.80  
Mass: 25.62 kDa  
  
 

| Sequence | MissedCleavage | Mass [Da] | wtIntensity | muIntensity | Score |
| --- | --- | --- | --- | --- | --- |
| EERPDVTLAGR | 1 | 1241.64 | 0.00 | 721.25 | 73.50 |
| GSSVSPLFPGGGVTFGPKPK | 1 | 1915.02 | 0.00 | 654.38 | 30.15 |
| GTQQLALK | 0 | 857.50 | 640.24 | 1170.63 | 79.12 |
|  |  |  | 640.2 | 2546.3 |  |
